# A Study on the Effect of Breed and Storage Temperature on Quality of Eggs Laid by Two Local Italian Hen Breeds

**DOI:** 10.3390/ani16121808

**Published:** 2026-06-11

**Authors:** Chiara Rizzi

**Affiliations:** Department of Agronomy, Food, Natural Resources, Animals and Environment (DAFNAE), University of Padova, Viale dell’Università 16, 35020 Legnaro, Italy; chiara.rizzi@unipd.it

**Keywords:** chicken biodiversity, local breeds, egg quality, storage, temperature

## Abstract

Among the ten local breeds of the Veneto region in Italy, Pepoi (PP) and Ermellinata di Rovigo (ER) hens start laying eggs earlier than the others. The egg laying rate (27–34 weeks of age) is higher (*p* < 0.01) in PP than in ER hens. Egg quality (at 33 weeks of age, 120 eggs/breed) was studied in fresh 1 day-old eggs and in preserved 21 day-old eggs according to breed and storage temperature (12 and 21 °C). Fresh ER eggs showed higher (*p* < 0.01) egg weights, yolk pH, Haugh units and yolk indices and lower (*p* < 0.01) eggshell lightness and thickness, surface area-to-volume ratios, and albumen pH than PP eggs, but the yolk-to-albumen ratio was similar between the breeds. After 21 days of storage, the egg traits showed the same trend for significant differences between breeds, with the exception of albumen pH and Haugh units, which were similar. Eggs stored at 21 °C showed lower (*p* < 0.01) Haugh units and yolk index values and higher (*p* < 0.01) albumen and yolk pH, albumen yellowness, and weight loss than eggs stored at 12 °C. Stored PP and ER eggs also differed in terms of observed changes in Haugh units, yolk pH and yolk index values with storage temperature: ER eggs showed higher (*p* < 0.01) yolk index values than PP eggs at both storage temperatures. Eleven weeks after the onset of laying, significant differences were observed in several traits of fresh and stored eggs from the studied breeds, particularly regarding the strength of the vitelline membrane. These preliminary results contribute to the characterization of the storage suitability of eggs from local breeds and to future crossbreeding programmes for enhancing chicken biodiversity.

## Simple Summary

Characterizing the yield performance and egg quality of local chicken breeds is essential for effective management of them and their products. Local breeds may exhibit different onsets of laying, and this may allow some breeds to anticipate the egg production period and extend the period of egg availability for market. Local breeds are currently reared outdoors; their products show seasonality and supply a niche market, as consumer interests are increasingly focused on the quality of both fresh and stored eggs. Among the ten breeds from the Veneto region in Italy, Pepoi (PP) and Ermellinata di Rovigo (ER) hens start laying eggs earlier than others. These breeds are dual-purpose (egg and meat) breeds, and PP hens are characterized by smaller adult bodies and egg sizes compared to ER birds. The breed and storage temperature (12 and 21 °C for 21 days) were found to affect many external and internal traits of fresh and stored eggs. Eggs preserved at 21 °C exhibited lower Haugh units and yolk index values, as well as higher albumen yellowness, than those stored at 12 °C; stored ER eggs exhibited higher yolk index values than PP eggs.

## 1. Introduction

The Veneto region in Northern Italy, situated on the Po plain, has a long-standing cereal cultivation tradition; this cereal provides the primary ingredients for poultry diets. This region exhibits considerable poultry biodiversity, with a total of ten chicken breeds, some of which have been recovered by means of breed recovery programmes. These breeds differ in origin, as well as in phenotype, productive purpose [1,2], yield performance and meat and egg quality [3,4,5]. These local breeds are currently reared outdoors on small farms, and their products supply a niche market [6]. Given the natural and non-intensive nature of their rearing production system, product availability is seasonal, in contrast to commercial hybrid strains, for which production is continuous. Some external egg traits are directly evaluated at market by consumers, particularly egg size and eggshell colour, which may affect purchasing choices, even though there is no correlation between the colour of the shell and internal traits [7]. The yolk-to-albumen ratio is important for the internal chemical composition of eggs [8] and their correct use in cooking; thus, it should be indicated on packaging, as it differs between breeds [4,9,10]. Internal changes in the albumen and yolk depend on many factors of endogenous and exogenous origin. Physical and chemical processes occur in the body of the hen according to its genotype and age [11], as well as its environmental rearing conditions [12]; these processes affect the egg components, starting from the ovary and oviduct [8,13]. Table eggs may be consumed up to 28 d after laying [14]; the duration of storage and the environmental conditions where the eggs are stored can affect internal egg quality [15,16], as well as eggshell quality [17]. Differences in storage conditions and times depend on the egg’s intended use: for table eggs, the refrigeration temperature should be about 4 °C for four weeks [18], whereas for hatching eggs, the optimal storage time is four days (up to seven days is also permissible) at a temperature of 14–18 °C and 70–75% relative humidity [19]. Most physical traits of preserved, intact eggs, such as the thickness of the albumen, the condition of the yolk vitelline membrane, and yolk shape, cannot be visually evaluated by the consumer [16]. Alongside these physical changes that can vary due to the storage duration and conditions, other physical and chemical changes occur between egg components [20], affecting the final quality of the egg.

Profiling the yield performance of purebreds and the quality of their products is an important step in the valorization of chicken biodiversity and the management and use of birds and their products. The aim of this study is to profile the quality of fresh and stored eggs of two chicken breeds from the Veneto region during the first phase of the laying cycle and to evaluate the effects of two storage temperatures on changes in the internal components of laid eggs.

## 2. Materials and Methods

### 2.1. Animals

**Figure 1:**
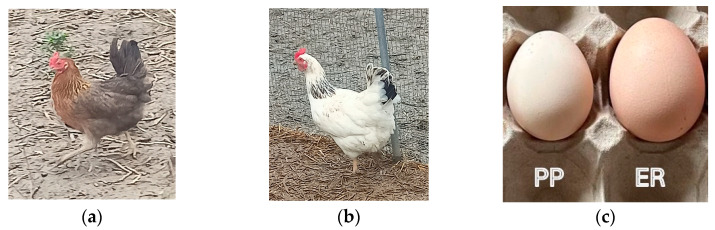
Pepoi—PP (**a**) and Ermellinata di Rovigo—ER (**b**) hens and eggs (**c**).

Two breeds were considered for the study: Pepoi (PP) and Ermellinata di Rovigo (ER). Hens belonging to these breeds exhibit different phenotypes, different coloured plumages, and lay eggs with different coloured eggshells (Figure 1). PP hens have an adult body weight of 1.3 kg, and ER hens have an adult body weight of 2.3 kg [1,2].

All the hens (60 hens/breed) used for this study were reared at the Center for Poultry Biodiversity Conservation in the Veneto region (Veneto Agricoltura, Ceregnano, Rovigo, Italy) in Northern Italy. The hens were reared outdoors; both breeds were maintained under the same environmental conditions from birth. Birds were reared under natural photoperiod conditions. Throughout the studied laying (October and November) and egg collection (November) periods, the photoperiod decreased (about from 12L:12D to 10L:14D) and the environmental temperature was 16.0 ± 4.1 °C (October) and 11.0 ± 3.2 °C (November). Each breed (60 hens) had free access to an outdoor space (5 m^2^/bird; equipped with linear drinkers), where the hens stayed throughout the day, and an indoor space (4 hens/m^2^; wood shavings and straw litter, with perches and circular feeders), mainly used at night and for laying eggs (collective nests); the areas (indoor and outdoor) available to each breed were divided by netting. Feed and water were provided ad libitum. The hens were fed a commercial pelleted layer feed (CP = 16%, ME = 11.6 MJ/kg, Ca = 3.2%, P = 0.6%).

### 2.2. Data Collection and Measurements

The hens started laying at about 22 weeks of age, and the eggs were collected at 33 weeks of age. The laying rate of each breed was monitored daily from 27 to 34 weeks; it was calculated as the total number of daily laid eggs/total number of hens × 100. The eggs (120 eggs/breed, excluding double-yolk eggs, dirty eggs, and eggs with shape imperfections or with cracks and toe holes in the shell) were collected according to European Regulations (EC No. 1/2005 and EC No. 1099/2009) on animal care and welfare. The egg sampling carried out in the Center for Poultry Biodiversity did not affect the welfare of the hens as it occurred when the animals were not in their nests, thus avoiding handling. The eggs were collected over eight consecutive days. The collected eggs were kept for 1 day at 15 °C and 65% relative humidity at the centre and then were transferred daily (1 h trip at 14 °C and 62% relative humidity) and randomly allocated to the following treatment groups: fresh eggs (1 d-old eggs) and stored eggs (kept for 21 days in rooms at two different temperatures and relative humidities (21 d-old eggs, stored at 12 °C and 72% relative humidity; 21 d-old eggs, stored at 21 °C and 62% relative humidity). During each daily collection, the eggs of each breed were numbered and weighed, and the external egg traits (eggshell colour, width and length) were measured. For each treatment group, each trait was measured for 30 eggs/breed. Measurements of egg weight, eggshell width and length, eggshell colour (L*, a*, b*), eggshell thickness, albumen weight, yolk weight, albumen pH, yolk pH, albumen height, yolk height and diameter, and yolk Roche colour were taken for fresh and stored eggs. For the eggs stored for 21 days, the albumen and yolk colour traits (L*, a*, b*) were also measured. The albumen was placed into a plastic tube (3 cm diameter) and white paper was placed beneath and surrounding it before colour measurement. The yolk colour was measured at the surface. After 21 days of storage, the eggs were weighed again, and the weight loss rate was calculated. To measure the weight of the eggs and their components, an electronic balance (0.01 g) was used. The eggshell, yolk and albumen colour were measured using a colorimeter (Chroma meter CR 300, Minolta Co, Ltd., Osaka, Japan), using the CIE scale [21]; L, a* and b* values reflect lightness (0 = black, 100 = white), redness (−100 = green, 100 = red) and yellowness (−100 = blue, 100 = yellow), respectively. The yolk colour was also measured using a DSM yolk fan (formerly the Roche scale and indicated as the Roche colour). The length (longitudinal axis) and maximum width (equatorial axis) of each egg were measured with callipers (0.01 mm), and the shape index was calculated as maximum width/length × 100. The surface area and the volume were calculated according to the formula [22]:

Surface area = (3.155 − 0.0136L + 0.0115W) LW,

Volume = (0.6057 − 0.0018W) LW^2^,

where L = egg length and W = egg maximum width.

The surface area-to-volume ratio was calculated as surface area/volume. The eggshell thickness (mineral layer with membranes) was measured at the equatorial region (wet eggshell, three measurements/egg) using digital callipers (0.001 mm) (Mitutoyo Co., Kawasaki, Japan). To determine the weight of the egg components, the yolk was manually separated from the albumen and weighed; the albumen weight was calculated as the difference between the weight of the egg and the sum of the weight of the yolk and eggshell (after drying at 50 °C per 12 h). After separating the albumen (thick and thin) and yolk of each egg, they were placed into plastic tubes, and pH values were measured using a pH meter (Hanna Instruments, Inc, Woonsocket, RI). The pH meter was calibrated using buffer solutions at pH 4 and 7 prior to measurements. The ratio of each egg component (yolk, albumen and eggshell) was calculated as the weight of each component/egg weight × 100, while the weight loss rate was calculated as (1 d-old egg weight—21 d-old egg weight)/1 d-old egg weight × 100. To calculate the Haugh units (HU) [23] and the yolk index (YI), each egg was weighed and broken, and the yolk and albumen were placed on a glass plate to measure the albumen and yolk heights and the yolk diameter. The thick albumen and the yolk height were measured using a tripod micrometre (0.01 mm) (Mitutoyo Co., Kawasaki, Japan), and the yolk diameter was measured at two points (near and far from chalaziferous layer) using callipers (0.01 mm). YI was calculated as the ratio between the yolk height and the average yolk diameter [24]. All measurements were carried out in a room with a temperature of 18 °C.

### 2.3. Statistical Analyses

Shapiro–Wilk and Levene’s tests were carried out on all data to check the normality of the data distribution and the homogeneity of variance between treatments, respectively (SAS 9.4, SAS Institute, Cary, NC, USA). Egg laying rates (from four biweekly periods and the total period) were evaluated through a two-way ANOVA (2 × 4) to assess the effect of breed, period and interaction. Data from 1 d-old eggs (albumen and yolk weight, yolk-to-albumen ratio, albumen and yolk pH, HU, YI, yolk Roche colour, yolk ratio, albumen ratio and eggshell ratio) was evaluated through 1-way ANOVA to assess the effect of breed. The initial (1 d) and final (21 d) quality traits (egg weight, eggshell colour, shape index, eggshell surface area-to-volume ratio, eggshell thickness, weight loss rate, albumen and yolk weight, albumen and yolk pH, albumen and yolk colour (L*, a*, b*), yolk Roche colour, yolk-to-albumen ratio, HU, YI, yolk ratio, albumen ratio and eggshell ratio) of stored eggs were evaluated through a two-way ANOVA (2 × 2), considering breed and storage temperature and their interaction as main effects. The proc GLM of SAS 9.4 (SAS Institute, Cary, NC, USA) was used. Significant differences among least square means were tested using Tukey’s test. Significance was set at *p* < 0.01 and *p* < 0.05.

## 3. Results

### 3.1. Egg Laying Rate

**Figure 2:**
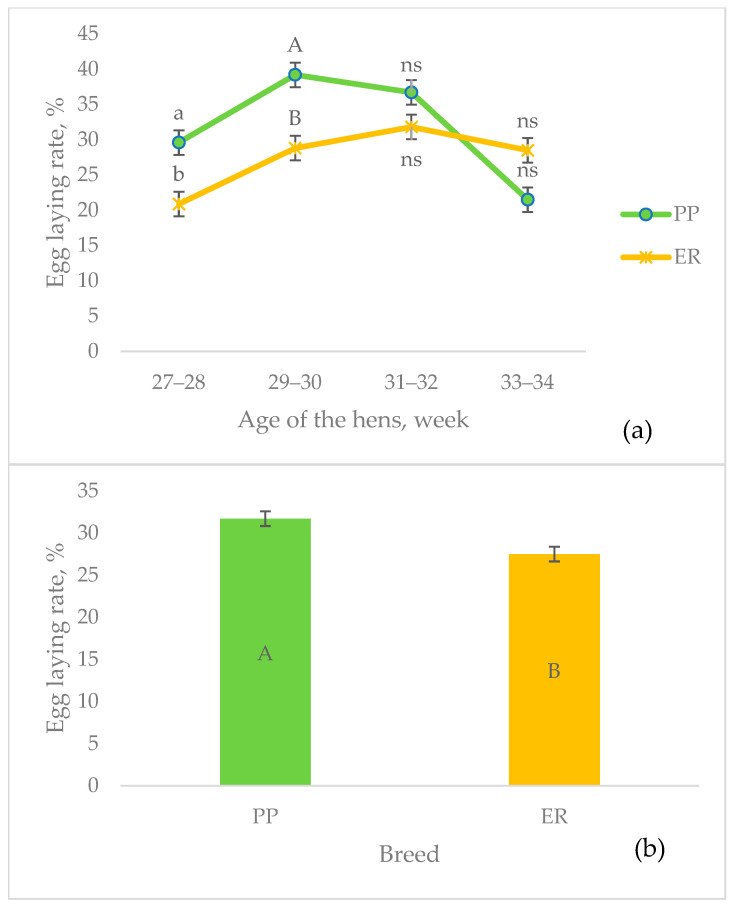
Effect of breed on egg laying rate (lsmeans ± SE) from 27 to 34 weeks of age, according to two-weekly (**a**) and total (**b**) periods. Breed: Pepoi = PP (green line and bar); Ermellinata di Rovigo = ER (orange line and bar). Different letters between lines and bars indicate different values. a, b: *p* < 0.05; A, B: *p* < 0.01. Observations (*n*): 14 for each biweekly period and 56 per total period, per breed.

The daily egg laying rates of the PP and ER hens are shown in Figure 2. At 27–28 (*p* < 0.05) and 29–30 (*p* < 0.01) weeks of age (Figure 2a), the two breeds exhibited different laying rates; that of PP was higher than that of ER. From 31 weeks to 34 weeks of age, the two breeds exhibited similar laying rates. Throughout the entire period (Figure 2b), the laying rate was higher (*p* < 0.01) in PP than in ER hens.

### 3.2. Egg Quality

#### 3.2.1. Fresh Eggs

In Table 1, the weight and eggshell traits of fresh eggs are shown.

The two local breeds lay eggs of different sizes, with PP eggs being smaller (*p* < 0.01) than ER eggs. Eggshell traits differed between the breeds, as PP showed a higher (*p* < 0.01) surface area-to-volume ratio and eggshell lightness (L*) and thickness. ER hens lay eggs with a tinted eggshell, showing higher (*p* < 0.01) values of redness (a*) and yellowness (b*) than those of PP. The egg shape index did not differ between the breeds.

Table 2 shows the effect of breed on the internal quality of fresh eggs.

ER eggs exhibited higher (*p* < 0.01) albumen and yolk weights than those of PP, while the yolk-to-albumen ratio was similar between the breeds. The albumen pH was higher (*p* < 0.01) in PP eggs, while the yolk pH was higher (*p* < 0.01) in ER eggs. The yolk Roche colour did not differ between the breeds, but the Haugh units and yolk index did differ, being higher (*p* < 0.01) in ER eggs.

**Figure 3:**
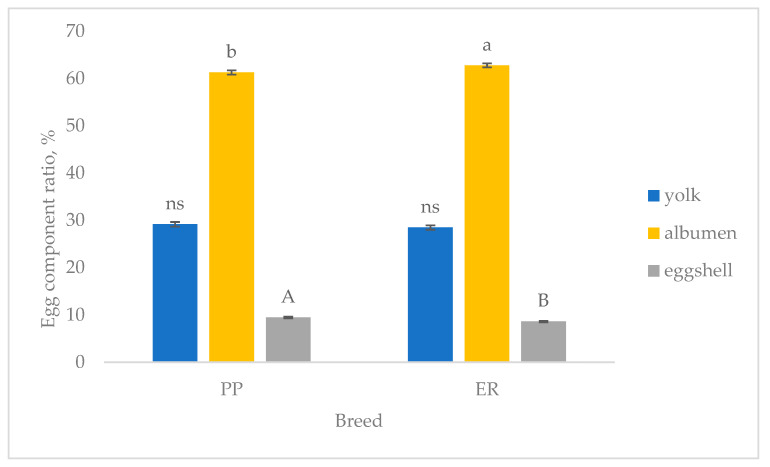
Effect of breed on the yolk, albumen and eggshell ratio (lsmeans ± SE) of 1 d-old eggs. Breed: Pepoi = PP; Ermellinata di Rovigo = ER. Different letters between bars of the same colour indicate different values. a, b: *p* < 0.05; A, B: *p* < 0.01. ns = not significant. Observations (*n*): 30 eggs per breed.

Figure 3 shows the component ratios in fresh eggs from both breeds.

In fresh eggs, the yolk ratio was similar between the breeds, while the albumen ratio was higher *(p* < 0.05) in ER eggs and the eggshell ratio was higher *(p* < 0.01) in PP eggs.

#### 3.2.2. Stored Eggs

Table 3 shows the effect of breed and storage temperature on the weight, weight loss and internal quality of the preserved eggs.

**Figure 4:**
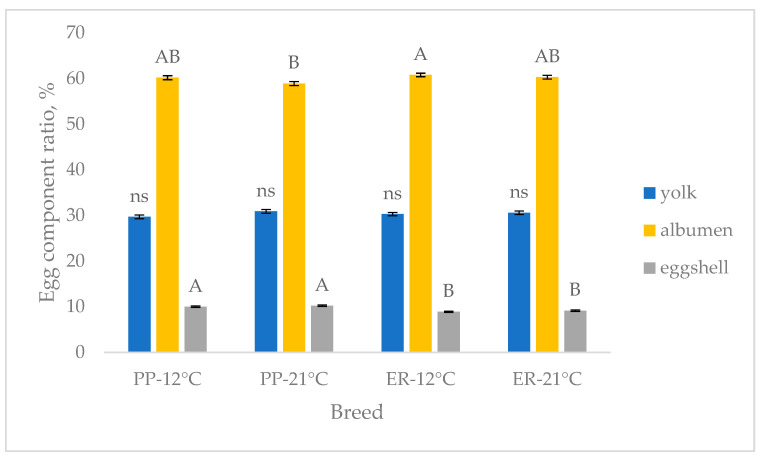
Effect of 21 d storage temperature on yolk, albumen and eggshell ratio (lsmeans ± SE) according to breed. Breed: Pepoi = PP; Ermellinata di Rovigo = ER. Different letters between bars of the same colour indicate different values. A, B: *p* < 0.01. ns = not significant. Observations (*n*): 30 eggs per breed and storage temperature.

After 21 days of storage, the final egg weight, similarly to the initial egg weight, differed between the breeds, being higher (*p* < 0.01) in ER eggs than in PP eggs. The initial and final egg weights were similar between the two storage temperatures. The rate of egg weight loss tended (*p* = 0.08) to be higher in PP than in ER eggs, whereas it was higher (*p* < 0.01) in the eggs preserved at 21 °C than in those preserved at 12 °C. ER eggs exhibited a higher (*p* < 0.01) albumen and yolk weight, yolk pH, yolk index and yolk Roche colour (*p* < 0.05) than PP eggs. They also exhibited higher (*p* < 0.01) albumen lightness and lower (*p* < 0.01) yolk lightness than PP eggs. The albumen pH, Haugh units, and yolk-to-albumen ratio were similar between the breeds. Eggs preserved at 21 °C exhibited lower albumen weights (*p* < 0.05), Haugh units (*p* < 0.01), and albumen redness (*p* < 0.05) and higher albumen pH (*p* < 0.01), albumen lightness (*p* < 0.05) and albumen yellowness (*p* < 0.01) than those preserved at 12 °C. Storage at 21 °C, when compared to that at 12 °C, increased the yolk pH (*p* < 0.01) and redness (*p* < 0.01), and decreased the yolk index (*p* < 0.01); the yolk weight, Roche colour, lightness and yellowness did not change. Stored eggs from both breeds (Figure 4) exhibited similar yolk ratios, whereas the albumen percentage differed between PP eggs stored at 21 °C and ER eggs stored at 12 °C, being higher (*p* < 0.01) in the latter. The eggshell ratio was higher (*p* < 0.01) in PP than in ER eggs at both storage temperatures.

Table 4 shows the changes in the internal quality of eggs stored at two different temperatures according to breed.

After 21 days of storage, the interaction effect was significant only for the yolk pH and the yolk index: the yolk pH was significantly (*p* < 0.01) different for ER eggs stored at 21 °C compared to all other temperatures and breeds, and the yolk index was significantly higher (*p* < 0.01) for eggs stored at 12 °C compared to those stored at 21 °C in both breeds. PP eggs exhibited a lower (*p* < 0.01) yolk index than ER eggs when stored at 12 °C. The weight loss increased (*p* < 0.01) according to storage temperature; it was similar between PP and ER eggs at both storage temperatures. The albumen pH differed between the two storage temperatures, being higher (*p* < 0.01) in 21 °C eggs than in 12 °C eggs, both for PP and ER. In PP, the Haugh units did not differ between eggs stored at 12 and 21 °C, whereas ER eggs stored at 21 °C exhibited lower (*p* < 0.01) Haugh units than those of eggs stored at 12 °C.

In Table 5, the effect of storage temperature on the colour traits of the albumen and yolk of preserved eggs is shown according to breed. No significant interaction effect was observed. Albumen lightness did not show differences between the two storage temperatures, neither in PP nor ER eggs. PP eggs stored at 12 °C showed the lowest (*p* < 0.01) L* values, while ER eggs stored at 21 °C showed the highest (*p* < 0.01). Albumen redness did not differ between groups, while albumen yellowness was higher (*p* < 0.01) in eggs stored at 21 °C than in those stored at 12 °C both for PP and ER. Yolk lightness did not change between the groups, while yolk redness significantly (*p* < 0.05) increased with the storage temperature, but only in ER eggs. The yellowness did not differ between the groups, and the yolk Roche colour did not change with storage temperature in both breeds.

## 4. Discussion

The Center for Biodiversity Conservation in Ceregnano, in the Veneto region, manages all ten local chicken breeds, from egg incubation and hatching to rearing of young birds and egg laying, over many months. According to the centre’s egg incubation programme, egg hatching, to obtain chicks of all breeds, occurs in the same month of the year in spring. Among the ten local breeds and coetaneous birds, two breeds show an earlier onset of laying than the others. The onset of laying depends on interactions between the birds’ physiological and bodily conditions and the environment [8,25]; the genetic asset that reflects, at least partially, the productive purpose of a breed may affect the pubertal age [25,26]. A chicken’s egg laying activity depends on the photoperiod [25,27]; under natural lighting conditions, the laying rate changes according to the season. In autumn, when the photoperiod is decreasing, it exhibits a negative trend. Among the ten local breeds, at the end of the summer season, when the birds were 6 months old, only PP and ER maintained laying rates ranging from 20 to 40% for many weeks, since at least the fourth week of production. This is an important aspect when evaluating a local breed, both for breeders and consumers. These breed characteristics may be considered in a breeding programme and for future crosses between genotypes, considering traits such as early body development and onset of laying. For purebreds, which are targeted at a niche market and are characterized by a slow body growth rate and egg production that are not competitive with those of hybrid genotypes [26], aspects like the seasonality and availability of marketable eggs may be strategic. These are the preliminary observations of these local breeds; more study is required to appropriately manage environmental factors such as the length of the photoperiod and the most appropriate diet according to breed. It should be noted that there is a lack of knowledge regarding the specific nutritional requirements of native purebred hens, given their different productive purposes and the interactions between genotype and environment [11,12]. According to egg marketing standards [14], PP eggs are small and ER eggs are medium in size. The egg weight is affected by the weight and size of the hen [11]; at the time of egg collection, the hens were at the beginning of the laying cycle, as laying started at about 5 months of age. The egg size changes according to breed, age at first egg, capacity of feed intake and ambient temperature [11]. At 33 weeks of age, the PP and ER eggs differed in weight and many eggshell traits; these differences were also characteristic of the eggs laid by older hens [4], with exception of the shape index. In the current trial, the shape index was similar between PP and ER eggs laid by young hens, while it differed in eggs laid by older hens, being higher in PP than in ER eggs [4]. Egg shape is strongly affected by the abdominal shape and oviduct morphology of the hen [28]; inhomogeneous variation in the properties of the eggshell double membrane and the pressure across the egg’s surface along the oviduct can give rise to different egg shapes [28]. In fresh eggs, an effect of breed on internal egg quality was evident for almost all the studied traits, whereas preserved eggs were less differentiated according to breed. The eggs preserved for 21 days, when compared to fresh eggs, showed differences in many traits, such as a drop in albumen weight, Haugh units and yolk index and an increase in albumen and yolk pH and yolk weight. The data regarding albumen and yolk weights indicate that, during storage, a portion of the albumen water evaporates through the eggshell, while another portion migrates into the yolk [20]. These changes affect the yolk-to-albumen ratio, the values of which varied from 0.46 to 0.51 between fresh and preserved eggs. Water passes from the albumen to the yolk after an egg is laid due to differences in osmotic pressure, and this continues during storage. In an ageing egg, an increase in albumen pH is a consequence of changes in the main component of its buffer system: H_2_CO_3_ is converted to water and carbon dioxide, which are partially released through the eggshell [29]. The eggshell cuticle, and factors affecting cover completeness [30], may slow down these processes and the natural decline in egg internal quality during storage [31]. An increase in the albumen pH destabilizes the integrity of the ovomucin–lysozyme complex, causing thick albumen thinning. The infiltration of some water from the albumen [29] increases the yolk pH. This passage of water may cause weakening of the vitelline membrane and a change in the yolk index [16]. Nevertheless, water from the egg yolk can also enter into the albumen, although it is still unclear why such migration occurs [20]. As previously observed, after 14 days of storage, free amino acids move from the yolk to the albumen and moisture moves from the albumen to the yolk, which may result in a decrease in yolk solid concentrations [29]. Marzec et al. [32] found that yolk viscosity is much more susceptible to the storage duration than the albumen quality traits, such as Haugh units and pH.

The storage temperature affects the rate of water exchange between the albumen and yolk, as well as between the albumen and eggshell [20]. In the present work, the temperature affected many internal egg processes, as shown by the significant differences between many traits of eggs preserved at 12 and 21 °C. The highest storage temperature significantly increased the albumen and yolk pH and significantly decreased Haugh units and yolk index, but more marked changes were observed in ER eggs. In these eggs, the yolk pH increased and the Haugh units decreased with storage temperature, whereas these traits were unchanged in PP eggs. Furthermore, PP eggs exhibited a lower yolk index than ER eggs, especially at 12°C. It is worth highlighting that PP eggs were much smaller than ER eggs, which can account for some of the differences in Haugh units and rates of changes in these values between the two breeds. As stated previously, yolk quality generally exhibits a higher rate of changes than the albumen [32]. Other authors have found that the decrease in egg quality under prolonged storage was greater in young hens than old hens [29]. For the albumen, the rate of quality deterioration during storage depended primarily on the initial albumen quality. Eggs with high Haugh units deteriorated more rapidly than eggs with low initial Haugh units; the albumen pH exhibits a higher rate of increase in eggs laid by younger hens than in those laid by older hens. The results of the present trial are consistent, partially, with previous reports from other authors, which described the effects of hen age and initial albumen traits on the quality of preserved eggs [29]. After storage, ER eggs showed a greater decrease in egg albumen quality than PP eggs; however, they exhibited a higher yolk index: the exact reasons for this response are unclear, even if the PP hens exhibited slightly earlier egg production and a higher egg laying rate than ER hens. Other authors found that that yolk index is generally lower in genotypes with higher egg production [32]. Consumers also assess egg internal quality via the properties of the yolk and albumen. The most important trait is the yolk colour, for both the pigment content and the capability to transfer it to cooking preparations, according to opinion [8]. The most perceivable colour changes between fresh and stored eggs occur in the albumen, although they depend on the storage conditions [20]. In the current trial, marked differences between PP and ER eggs were observed in albumen and yolk lightness, with opposite trends, although it is unknown which changes occurred in the structure and composition of albumen, vitelline membrane and yolk. The highest storage temperature positively affected the albumen b* index and lightness and negatively affected the a* index. According to previous findings [20], a more marked “yellowing” phenomenon is observed in the albumen of eggs stored at a higher temperature. The increased yellowness index indicates chemical changes involving colour traits. Among the causes of a deepening, darkening albumen and yolk colour are the Maillard reaction, which produces brown or even black melanoids, and a decrease in moisture, which leads to pigment accumulation [20]. Given the range of temperatures considered in the current study and that colour changes mainly significantly affected the albumen, it seems that the most probable reason is moisture loss, mainly in the albumen, which was higher in eggs stored at 21 °C. The weight loss rate is an important index reflecting the quality change in eggs during storage. Under the same environmental conditions, it may be affected by many factors, including eggshell traits, such as eggshell thickness and macro and ultra structures, and also by albumen and yolk quality [20]. Other than the sole thickness, the eggshell surface area-to-volume ratio and structure may affect the weight loss and other egg internal processes [15,33]. ER eggs, although having a thinner eggshell than PP eggs, tended to exhibit lower weight losses and a higher yolk quality, even at the highest storage temperature. Eggshell thickness changes according to many environmental factors, but it also changed with breed and age [34]. The ER eggshell is characterized by a lower thickness than other local breeds in the Veneto region, even for older hens [4]. More studies are needed to elucidate the role of the main egg components in the changes in egg quality during storage for these breeds.

## 5. Conclusions

Given the importance of biodiversity, all the productive traits of local chicken breeds, which are characterized by a notably lower yield performance than hybrid hens, should be analyzed in depth according to the environmental conditions where the hens are reared to determine suitable future management and distribution practices for these genotypes. Among the ten local chicken breeds of the Veneto region, only PP and ER hens start laying earlier than coetaneous hens of other breeds, and they produce marketable eggs after a few weeks of production. This trial aims to highlight some properties of egg production in Italian chicken purebreds from the Veneto region. Characterizing the yield performance and egg quality of these breeds will allow for identification of the most appropriate rearing and management conditions for these birds and their products. Knowledge of the yield performance and quality profile of fresh and stored eggs of these local breeds should be translated to future breeding programmes and to consumers to help them make informed choices related to niche products.

## Figures and Tables

**Table 1:** Effect of breed on the egg weight and eggshell traits of 1 d-old eggs.

	PP ^1^	ER ^1^	*p-*Value	RMSE
Egg weight, g	44.4 ^B^	53.3 ^A^	<0.0001	3.9986
Egg shape index	74.4	74.5	0.1655	3.3311
Surface area/volume	1.40 ^A^	1.32 ^B^	<0.0001	0.0375
L*	85.7 ^A^	71.6 ^B^	<0.0001	2.7705
a*	1.62 ^B^	10.7 ^A^	<0.0001	1.7271
b*	15.7 ^B^	23.7 ^A^	<0.0001	2.4367
Eggshell thickness, µm	328 ^A^	313 ^B^	0.0151	27.2428

^1^ Pepoi = PP; Ermellinata di Rovigo = ER. Different letters in the same row indicate different values. A, B: *p* < 0.01. RMSE = Root Mean Square Error. Observations (*n*): 60 eggs per breed.

**Table 2:** Effect of breed on albumen and yolk traits of 1 d-old eggs.

	PP ^1^	ER ^1^	*p-*Value	RMSE
Albumen weight, g	26.9 ^B^	32.5 ^A^	<0.0001	2.3997
Albumen pH	9.14 ^A^	9.02 ^B^	0.0038	0.0954
Haugh units	97 ^B^	103 ^A^	<0.0001	4.3899
Yolk weight, g	12.7 ^B^	14.8 ^A^	0.0002	1.4201
Yolk pH	6.41 ^B^	6.74 ^A^	0.0014	0.2235
Yolk index	0.506 ^B^	0.581 ^A^	<0.0001	0.0311
Yolk Roche colour	8.87	9.39	0.0801	0.9183
Yolk/albumen	0.481	0.455	0.0931	0.0427

^1^ Pepoi = PP; Ermellinata di Rovigo = ER. Different letters in the same row indicate different values. A, B: *p* < 0.01. RMSE = Root Mean Square Error. Observations (*n*): 30 eggs per breed.

**Table 3:** Effect of breed and storage temperature on weight, weight loss and internal quality traits of 21 d-old eggs.

	Breed ^1^			Temperature ^2^			
	PP	ER	*p*-Value	12 °C	21 °C	*p-*Value	RMSE
Initial egg weight, g	44.4 ^B^	53.3 ^A^	<0.0001	48.8	48.9	0.8827	3.9986
Final egg weight, g	43.4 ^B^	52.1 ^A^	<0.0001	48.4	47.1	0.1658	3.9631
Egg weight loss, %	2.35	2.17	0.0845	0.875 ^B^	3.64 ^A^	<0.0001	0.5010
Albumen weight, g	25.8 ^B^	31.6 ^A^	<0.0001	29.3 ^a^	28.1 ^b^	0.0421	2.7107
Albumen pH	9.32	9.30	0.0657	9.26 ^B^	9.36 ^A^	<0.0001	0.0474
Haugh units	71.8	73.4	0.4719	76.6 ^A^	68.7 ^B^	0.0006	10.4071
Albumen L*	29.08 ^B^	30.3 ^A^	0.0107	29.2 ^b^	30.1 ^a^	0.0412	2.3779
Albumen a*	−3.68	−3.37	0.2114	−3.23 ^a^	−3.81 ^b^	0.0278	1.3593
Albumen b*	6.81	7.26	0.3025	5.55 ^B^	8.52 ^A^	<0.0001	2.2652
Yolk weight, g	13.2 ^B^	16.0 ^A^	<0.0001	14.7	14.5	0.5520	1.3532
Yolk pH	6.43 ^B^	6.62 ^A^	<0.0010	6.38 ^B^	6.66 ^A^	<0.0001	0.2576
Yolk index	0.467 ^B^	0.514 ^A^	<0.0001	0.524 ^A^	0.458 ^B^	<0.0001	0.0307
Yolk/albumen	0.510	0.504	0.5160	0.497 ^b^	0.517 ^a^	0.0455	0.0470
Yolk L*	56.9 ^A^	54.8 ^B^	0.006	56.0	55.7	0.7378	3.8106
Yolk a*	1.77	2.19	0.3414	1.33 ^B^	2.63 ^A^	0.0044	2.3299
Yolk b*	43.0	41.9	0.1415	42.9	41.9	0.1663	3.7923
Yolk Roche colour	9.65 ^b^	10.2 ^a^	0.0280	9.89	9.98	0.7463	1.2014

^1^ Pepoi = PP; Ermellinata di Rovigo = ER. ^2^ Storage temperature. Different letters in the same row, for each treatment, indicate different values. a, b: *p* < 0.05; A, B: *p* < 0.01. RMSE = Root Mean Square Error. Observations (*n*): 60 eggs per breed and storage temperature.

**Table 4:** Effect of 21 d storage temperature on egg weight loss and albumen and yolk quality according to breed.

	PP ^1^		ER ^1^			
	12 °C	21 °C	12 °C	21 °C	*p-*Value	RMSE
Egg weight loss, %	0.893 ^B^	3.809 ^A^	0.858 ^B^	3.480 ^A^	0.1613	0.5010
Albumen pH	9.27 ^B^	9.37 ^A^	9.25 ^B^	9.35 ^A^	0.6165	0.0474
Yolk pH	6.34 ^B^	6.52 ^B^	6.42 ^B^	6.81 ^A^	0.0467	0.2576
Haugh units	74.9 ^AB^	68.8 ^AB^	78.3 ^A^	68.6 ^B^	0.4276	10.4071
Yolk index	0.490 ^B^	0.445 ^C^	0.557 ^A^	0.471 ^BC^	0.0024	0.0307

^1^ Pepoi = PP; Ermellinata di Rovigo = ER. Different letters in the same row indicate different values. A, B, C: *p* < 0.01. RMSE = Root Mean Square Error. Observations (*n*): 30 eggs per breed and storage temperature.

**Table 5:** Effect of 21 d storage temperature on albumen and yolk colour according to breed.

	PP ^1^		ER ^1^			
	12 °C	21 °C	12 °C	21 °C	*p-*Value	RMSE
Albumen						
L*	28.7 ^B^	29.4 ^AB^	29.6 ^AB^	30.8 ^A^	0.5854	2.3779
a*	−3.11	−3.62	−3.35	−4.00	0.7791	1.3593
b*	5.81 ^B^	8.70 ^A^	5.28 ^B^	8.34 ^A^	0.8406	2.2652
Yolk						
L*	56.6	57.1	55.3	54.3	0.2707	3.8106
a*	1.32 ^b^	2.21 ^ab^	1.34 ^b^	3.05 ^a^	0.3664	2.3299
b*	42.5	41.3	43.4	42.5	0.8596	3.7923
Yolk Roche colour	9.05 ^b^	9.70 ^ab^	9.79 ^a^	10.3 ^a^	0.6816	1.2014

^1^ Pepoi = PP; Ermellinata di Rovigo = ER. Different letters in the same row indicate different values. a, b: *p* < 0.05; A, B: *p* < 0.01. RMSE = Root Mean Square Error. Observations (*n*): 30 eggs per breed and storage temperature.

## Data Availability

The data presented in this study will be made available by the author on request (the datasets presented in this research are part of an ongoing study).
